# Increased Leaf Nicotine Content by Targeting Transcription Factor Gene Expression in Commercial Flue-Cured Tobacco (*Nicotiana tabacum* L.)

**DOI:** 10.3390/genes10110930

**Published:** 2019-11-14

**Authors:** Hai Liu, Tatyana I. Kotova, Michael P. Timko

**Affiliations:** Department of Biology, University of Virginia, Charlottesville, VA 22904, USA; hl9h@virginia.edu (H.L.); tik7c@virginia.edu (T.I.K.)

**Keywords:** nicotine, transcription factor (TF), ethylene response factor (ERF), tobacco

## Abstract

Nicotine, the most abundant pyridine alkaloid in cultivated tobacco (*Nicotiana tabacum* L.), is a potent inhibitor of insect and animal herbivory and a neurostimulator of human brain function. Nicotine biosynthesis is controlled developmentally and can be induced by abiotic and biotic stressors via a jasmonic acid (JA)-mediated signal transduction mechanism involving members of the APETALA 2/ethylene-responsive factor (AP2/ERF) and basic helix-loop-helix (bHLH) transcription factor (TF) families. AP2/ERF and bHLH TFs work combinatorically to control nicotine biosynthesis and its subsequent accumulation in tobacco leaves. Here, we demonstrate that overexpression of the tobacco NtERF32, NtERF221/ORC1, and NtMYC2a TFs leads to significant increases in nicotine accumulation in T2 transgenic K326 tobacco plants before topping. Up to 9-fold higher nicotine production was achieved in transgenics overexpressing NtERF221/ORC1 under the control of a constitutive GmUBI3 gene promoter compared to wild-type plants. The constitutive 2XCaMV35S promoter and a novel JA-inducible 4XGAG promoter were less effective in driving high-level nicotine formation. Methyljasmonic acid (MeJA) treatment further elevated nicotine production in all transgenic lines. Our results show that targeted manipulation of NtERF221/ORC1 is an effective strategy for elevating leaf nicotine levels in commercial tobacco for use in the preparation of reduced risk tobacco products for smoking replacement therapeutics.

## 1. Introduction

Pyridine alkaloids are toxic compounds that play a key role in plant defense mechanisms against herbivore and insect attack [1,2,3,4]. In cultivated tobacco (*Nicotiana tabacum* L.), nicotine usually accounts for about 90% of the total alkaloids, with nornicotine, anabasine and anatabine comprising the majority of the remaining alkaloid content [5]. During the natural growth cycle, and in the absence of significant biotic or abiotic stress, cultivated tobacco plants produce only minimal basal levels of nicotine due to the high cost of metabolism. However, this level becomes elevated rapidly in response to insect or animal herbivory or in response to wounding, triggered by natural or human-mediated topping or decapitation [6,7,8]. The decapitation or wounding response is well known to result in the induced biosynthesis and transportation of jasmonic acid (JA) and its derivatives, such as methyljasmonic acid (MeJA), as part of a damage signal from shoot to root to promote the biosynthesis of nicotine and other alkaloids [9,10]. The consequence is rapid nicotine biosynthesis.

Nicotine is exclusively synthesized in the roots of tobacco, subsequently translocated to aerial parts of the plant via xylem, and finally mobilized into the central vacuoles of leaf mesophyll cells mediated by the multidrug and toxic compound extrusion (MATE) transporters [6,7,11,12,13]. Over the past several decades, genes encoding the enzymes in the nicotine biosynthetic pathway have been identified and characterized allowing a greater understanding of how the pathway works [4,14,15,16] (see also Supplementary Figure S1).

Nicotine is formed enzymatically through the condensation of nicotinic acid (pyridine ring) and *N*-methyl-Δ^1^-pyrrolinium cation (pyrrolidine ring) [17]. The formation of the pyrrolidine ring starts with the conversion of the diamine putrescine to *N*-methylputrescine by putrescine *N*-methyltransferase (PMT), with putrescine being formed from arginine and ornithine by arginine decarboxylase (ADC) and ornithine decarboxylase (ODC) [18,19,20,21]. *N*-methylputrescine is then oxidized and cyclized to form *N*-methyl-Δ^1^-pyrrolinium cation by *N*-methylputrescine oxidase (MPO) [22,23]. The pyridine ring derived from aspartate involves the biosynthesis of nicotinic acid dinucleotide (NAD) controlled by aspartate oxidase (AO), quinolinate synthase (QS), and quinolinic acid phosphoribosyltransferase (QPT) [24,25,26]. The final nicotine ring coupling is mediated by the action of a PIP-family isoflavone reductase-like enzyme (A622) and a berberine bridge enzyme-like enzyme (BBL), although the exact reaction mechanism has yet to be fully elucidated [27,28,29].

The inductive regulation of nicotine biosynthesis is a complex mechanism involving several levels of signal transduction and transcriptional regulation [4]. Convincing evidence has shown that JA induces transcriptional upregulation of a suite of genes involved in nicotine biosynthesis and involves members from at least two distinct transcription factor families, the AP2 domain-containing ethylene response factor (ERF) gene family and the MYC2-like basic helix-loop-helix (bHLH) gene family [30,31,32,33]. Two JA-responsive ERFs, ERF221/ORC1 and ERF10/JAP1, have been identified in tobacco and shown to upregulate the expression of *PMT*, the key enzyme in nicotine biosynthesis [30]. In 2008, the phylogenetic relationship among members of the tobacco AP2/ERF superfamily was reported and members of the Group IX clade were identified as main regulators of JA-induced responses [31]. A cluster of seven Group IX members of the ERF superfamily were subsequently shown to constitute the *NIC2*-locus, a well-documented major regulator of alkaloid formation in tobacco, and these ERFs were demonstrated to function by binding to a GCC-box element in the promoter region of the genes [21,32,34,35,36,37]. Subsequently, a non-*NIC2* locus tobacco ERF, ERF32, was identified and demonstrated to also independently positively regulate JA-induced nicotine biosynthesis in BY-2 cells [35].

The specific recognition of (+)−7-iso-Jasmonoyl-L-isoleucine (JA-Ile), the bioactive form of JA, by the F-box protein CORONATINE INSENSITIVE 1 (COI1) in the Skp1–Cul1–F-box protein (SCF) ubiquitin E3 ligase complex leads to the formation of a stable COI1/JA-Ile complex that binds to the JASMONATE ZIM DOMAIN (JAZ) transcriptional repressors, resulting in their ubiquitination and subsequent degradation by the 26S proteasome [38,39]. In Arabidopsis, removal of the JAZ repressor, which is also associated with the co-repressor TOPLESS (TPL) by NOVEL INTERACTOR of JAZ (NINJA), releases the bHLH family MYC2/3 proteins for transcriptional activation of downstream targets [40,41,42,43]. Recently, a subunit of the Arabidopsis Mediator complex, MEDIATOR25 (MED25), has been proven to positively regulate JA signaling through interaction with COI1 and MYC2 in the promoter regions of MYC2 target JA-responsive genes [44,45]. A similar regulatory framework also exits in tobacco, where a small gene family of NtJAZ repressors has been characterized and in vivo evidence confirmed the interactions between specific NtJAZ and NtMYC family members in the nucleus leading to the regulation of nicotine biosynthetic gene expression in response to JA [35,46,47]. It is also expected that similar Med25-MYC2 activation of JA signaling exists in tobacco, since the mediator is considered as a highly conserved co-activating complex in a wide range of eukaryotes [44,48,49,50].

It has also been well established that the transactivation of nicotine biosynthetic gene expression in tobacco involves NtMYC1/2 and NtERF transcription factors (TFs) coordinated through specific cis-regulatory sites in the proximal promoter regions of the structural genes responsible for nicotine biosynthesis [21,32,46,51]. These G-box and GCC-box elements are essential for proper regulation and variation, both in the sequence of the conserved core and flanking regions as well as the exact member of the two TF families binding them fine tunes the system to control expression levels [37,52]. Silencing of NtMYC2 gene expression using RNA interference (RNAi) constructs indicated that NtMYC may also directly regulate the transcription of related NtERFs [51] (also summarized in Supplementary Figure S2). Similarly, expression suppression of the *NIC2*-locus ERF genes compromises induction of the nicotine biosynthetic genes [32,33,46].

Substantial evidence has accumulated indicating that it is possible to alter the levels of nicotine and related alkaloids either by modification of the expression of genes encoding the biosynthetic enzymes or the transcription factors that are involved in regulating them. Most of these studies focused on the knock-down or complete repression of expression of genes in the biosynthetic pathway and were aimed at reducing or eliminating nicotine from the plant [27,28,53,54,55,56,57]. A few studies targeted regulatory factors to achieve this end [35,36,46]. The extent to which mis-regulation of nicotine biosynthetic gene expression or the transcriptional regulatory factors that control this expression can be used to elevate nicotine content in the leaves of commercial tobacco plants growing in the field either under physiological conditions or following topping is unclear. While in the past, elevated nicotine was an undesirable characteristic, with the increased movement toward nicotine-based products that can effectively substitute for cigarettes but with very low risks, there is a need for source material for the production of naturally synthesized nicotine and leaf extracts that can be used in the formulation of reduced risk products (RRPs, e.g., electronic cigarette and vapor products, heat-not-burn devices, etc.) [58,59]. There is also emerging research that suggest that nicotine and other drugs that act as nicotinic acetylcholine receptors (nAChRs) may be beneficial in the management of Parkinson’s disease, Alzheimer’s, and related neurological disorders, making the large-scale production of highly purified nicotine at low costs for therapeutics necessary [60,61].

In the present study, we examined the potential for directed modification of TF abundance as a means for elevating nicotine levels in tobacco plants. We used two constitutive gene promoters and a novel synthetic JA-responsive gene promoter to provide feed forward potentiation of nicotine expression. Through a systematic, parallel comparison of various transgenes, we demonstrate that transgenic overexpression of NtERF221 significantly increases nicotine levels in commercial varieties prior to topping, achieving levels substantially greater than in decapitated (i.e., topped) plants, and these levels can be further elevated by treatment with JA. Therefore, targeted manipulation of NtERF221 may be an effective strategy for the elevation of nicotine levels in the leaves of commercial tobacco for later use in preparation of extracts and cured leaf materials for use in reduced risk tobacco products and therapeutics.

## 2. Materials and Methods

### 2.1. Plant Materials and Transformation

Seeds of *N*. *tabacum* L. variety K326 were obtained from the US National Plant Germplasm System (NPGS) and grown in the greenhouses at the University of Virginia. Seeds and plants used in this study were maintained disease and pest free. Wild-type seeds were germinated on agar plates (100 × 20 mm) containing basic Murashige and Skoog (MS) media [62] at 25 °C under 12 h light/12 h dark photoperiods. For quantitative reverse-transcription polymerase chain reaction (qRT-PCR), germinated small seedlings were transferred onto larger MS plates (120 × 17 mm) and grown vertically until two weeks old, before being treated with 0.1% dimethyl sulfoxide (DMSO) (control) or 100 µM MeJA by spray. For thin layer chromatography (TLC) and gas chromatography-mass spectrometry (GC-MS) analysis, germinated seedlings were transferred in soil and grown under the same condition until five weeks old before DMSO or MeJA treatment.

*Agrobacterium tumefaciens* strain LBA4404 was used to introduce the various transgenes into tobacco [63]. T0 transgenic plants recovered from cell culture were confirmed as harboring the transgene by genomic DNA PCR. The T0 plants were then self-pollinated and the resulting T1 seeds germinated on agar plates (100 × 20 mm) and contained on MS plates supplemented with 50 mg/L hygromycin. A rapid screen using TLC was employed to identify high nicotine producers which were also verified by qRT-PCR. The promising T1 lines were maintained in the greenhouse and self-pollinated to produce T2 generation seeds. The non-segregating T2 tobacco seedlings and plantlets were used for further study on gene expression and nicotine quantification.

### 2.2. DNA Cloning and Vector Construction

Coding regions (CDS) of *NtERF10* (CQ808845), *NtERF32* (AB828154), *NtERF121* (AY655738), *NtERF221* (CQ808982), *NtMYC2a* (HM466974), *NtPMT1a* (AF126810), *NtQPT2* (AB038494), and *NtA622* (D28505) were amplified by PCR with Phusion High-Fidelity DNA Polymerase (New England Biolabs, Ipswich, MA, USA) and introduced into Gateway pDONR221 vector via BP recombination reaction for sequence verification. The PCR-amplified promoter sequence of *Glycine max Ubiquitin-3* (*GmUBI3*) gene and artificially synthesized 4XGAG promoter derived from *NtPMT1a* gene were used to replace the original dual cauliflower mosaic virus (CaMV) 35S promoter in the binary vector pMDC32, namely pGmUBI3-MDC and p4GAG-MDC, for Gateway compatibility. Sequence-verified genes in pDONR221 were then sub-cloned into pMDC32, pGmUBI3-MDC, and p4GAG-MDC, respectively. The resulting constructs were designated as 35S:ERF10, 35S:ERF32, 35S:ERF121, 35S:ERF221, 35S:MYC2a, 35S:PMT1a, 35S:QPT2, 35S:A622, GmUBI3:ERF10, GmUBI3:ERF32, GmUBI3:ERF121, GmUBI3:ERF221, GmUBI3:MYC2a, GmUBI3:PMT1a, GmUBI3:QPT2, GmUBI3:A622, 4GAG:ERF10, 4GAG:ERF32, 4GAG:ERF121, 4GAG:ERF221, 4GAG:MYC2a, 4GAG:PMT1a, 4GAG:QPT2, and 4GAG:A622.

### 2.3. RNA Isolation and Quantitative Reverse-Transcription Polymerase Chain Reaction (qRT-PCR)

For gene expression analysis, five to six two-week-old whole seedlings were collected together for each RNA isolation and three independent RNA isolations were performed for each sample. Total RNA was isolated with TRIzol reagent (ThermoFisher Scientific, Waltham, MA, USA) following the manufacturer’s instructions. DNA contaminants were removed from total RNA with RNase-free DNase I (New England Biolabs, Ipswich, MA, USA). The DNA-eliminated total RNA was then reverse-transcribed using a QuantiTect reverse transcription kit (Qiagen, Hilden, Germany). Quantitative PCR was conducted using iTaq^™^ Universal SYBR^®^ Green Supermix (Bio-Rad Laboratories, Hercules, CA, USA) on an CFX96^™^ Real-Time PCR detection system (Bio-Rad Laboratories, Hercules, CA, USA). The relative expression level of each gene was normalized to the level of *N*. *tabacum Elongation Factor 1-alpha* (*NtEF-1α*) [64]. An unpaired *t*-test was then performed to compare each sample to its relative control under the same treatment group.

### 2.4. Alkaloid Extraction and Thin Layer Chromatography (TLC)

Alkaloid extraction was performed as described by Goossens et al. [65]. Briefly, five-week-old wild-type and transgenic plantlets were sprayed with 0.1% DMSO (control) or 100 µM MeJA. Leaves were then collected and lyophilized 48 h after the treatment. 50 mg lyophilized tissue were homogenized in liquid nitrogen and basified with 10% NH_4_OH. 100 µg quinaldine was added as an internal standard. Total alkaloids were extracted with CH_2_Cl_2_, vacuum concentrated, and resuspended into 400 µL CH_2_Cl_2_. For the TLC assay, alkaloid extracts from three individuals of each line were mixed together and then equal amounts of the extracts from different lines were loaded onto a silica gel TLC plate (UV254, Analtech). Separation was done with the mobile phase composed of dichloromethane: methanol: 10% NH4OH (125:15:2). Spots were visualized by the spray with Dragendorff reagent (Sigma-Aldrich, St. Louis, MO, USA).

### 2.5. Gas Chromatography-Mass Spectrometry (GC-MS) Measurement of Nicotine

For GC-MS analysis of nicotine, alkaloids were extracted with naphthalene-d8 as an internal standard. For each transgenic line, total alkaloids were separately extracted from six to eight five-week-old individuals. For each transgenic construct, three independent lines were assayed. Nicotine concentration was measured on a Shimadzu GCMS QP2010 plus system (Shimadzu, Kyoto, Kyoto Prefecture, Japan) with a protocol developed previously [46,65]. The statistical test was performed by the analysis of variance (ANOVA) followed by Tukey’s honest significant difference (Tukey’s HSD) test using R (version 3.4.4).

## 3. Results

### 3.1. Constitutive and Inducible Overexpression of Transcription factors NtERF32, NtERF221, and NtMYC2a Increases Nicotine Contents

**Figure 1:**
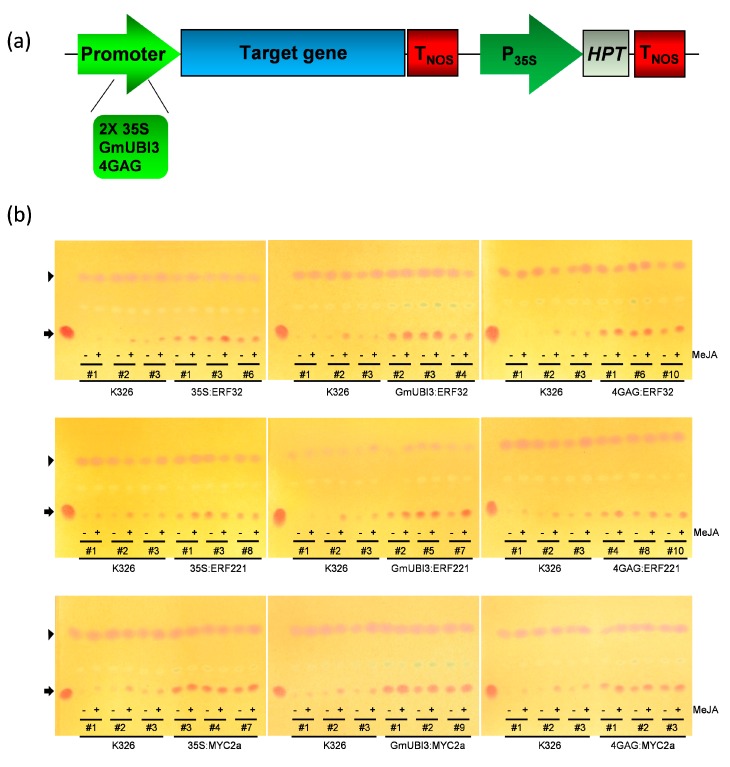
Schematic of vector construction and thin layer chromatography (TLC) analysis of nicotine in wild-type and transgenic tobacco. (**a**) Schematic of the binary vector construction used for overexpression in tobacco. (**b**) TLC assay for the detection of nicotine accumulation in the leaves of 5-week-old wild-type and the *NtERF32*, *NtERF221*, or *NtMYC2a* overexpression lines. The plantlets were treated with 0.1% DMSO (control) or 100 µM methyljasmonic acid (MeJA) for 48 h before the leaf tissue was collected for the alkaloid extraction. Arrows indicate nicotine bands, and arrowheads indicate quinaldine as internal control.

The expression of genes encoding the biosynthetic enzymes involved in nicotine formation is highly regulated in the roots of wild and cultivated tobacco, and both AP2/ERF and bHLH TFs have been shown to be involved in regulation of these genes (Supplementary Figure S2). In order to determine if the overexpression of one or more specific TF is an effective means of increasing nicotine levels in flue-cured tobacco (*Nicotiana tabacum* L.), we transformed K326 plants (a typical cultivated variety) with a set of transgene constructs, in which the coding sequences of five TF genes previously implicated in controlling nicotine biosynthetic gene expression (i.e., *NtERF10*, *NtERF32*, *NtERF121*, *NtERF221* and *NtMYC2a*) were placed under the control of either a double-enhanced CaMV 35S promoter (2X35S) known to provide a high-level constitutive expression, a soybean *Ubiquitin-3* gene promoter (GmUBI3) capable of directing very high levels of constitutive expression in tobacco [66], or a novel JA-inducible promoter comprised of four copies of the GAG regulatory motif linked in tandem upstream of a minimal promoter originating from the tobacco *NtPMT1a* gene (4XGAG). This promoter gives high-level root-specific and JA-regulated expression consistent with the timing and location of alkaloid formation [21,35] (Figure 1a).

Transgenic K326 tobacco lines were generated by Agrobacterium-mediated transformation and at least eight independent T0 lines (primary transformants) were generated per construct. T0 plants were progressed to the T1 generation by self-fertilization and hygromycin-resistant T1 individuals confirmed to have intact transgenes present in their genome were subject to further analysis. Transgenic T1 lines were sprayed with either 0.1% DMSO (mock treatment) or 100 µM MeJA, and TLC analysis of leaf tissue extracts showed that transgenic lines overexpressing the three different TF genes (i.e., *NtERF32*, *NtERF221* and *NtMYC2a*) had significantly elevated levels of nicotine in their leaves relative to wild-type K326 plants (Figure 1b). These promising lines were maintained and self-pollinated to give T2 seeds for further detailed analysis.

**Figure 2:**
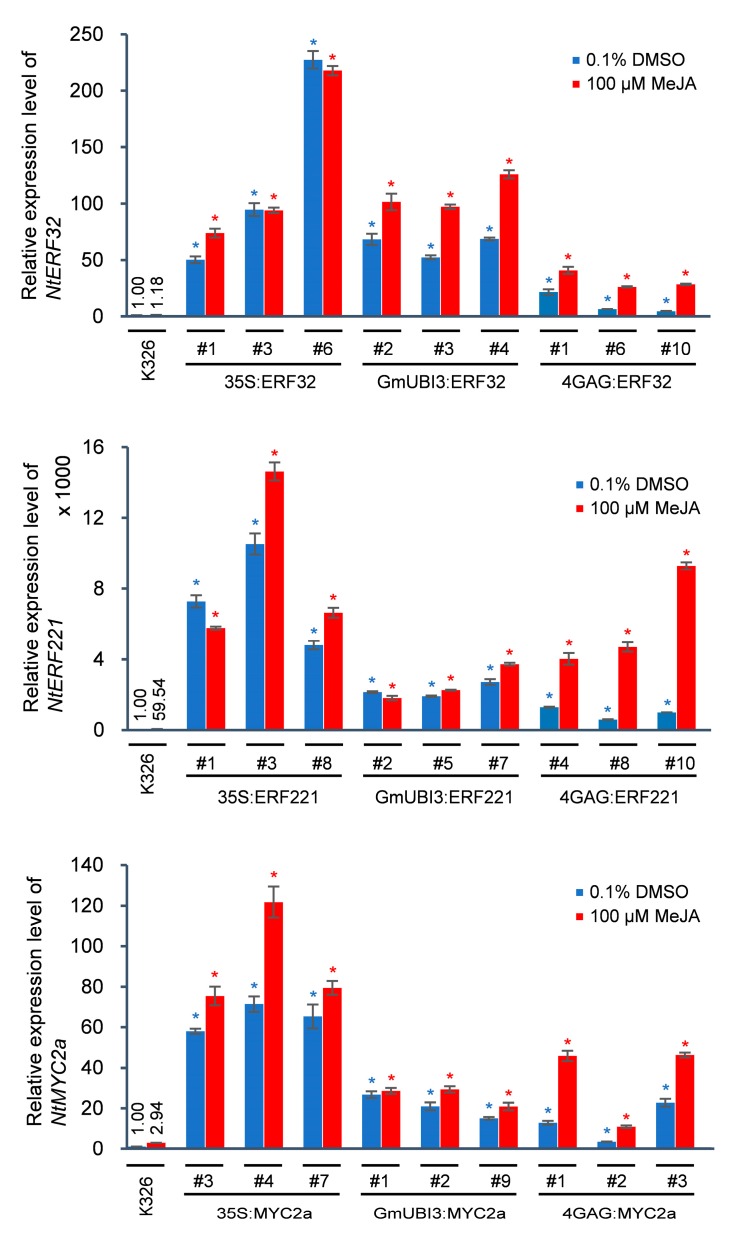
Quantitative reverse-transcription polymerase chain reaction (qRT-PCR) verification of the transcript levels of *NtERF32*, *NtERF221*, and *NtMYC2a* in wild-type and transgenic tobacco. Two-week-old wild-type or T2 generation seedlings of *NtERF32*, *NtERF221* and *NtMYC2a* overexpression lines were treated with 0.1% DMSO (control) or 100 µM MeJA for 8 h before being collected for total RNA extraction and qRT-PCR experiment. Expression level was represented as mean of relative expression values from three biological replicates (*n* = 3) normalized to *N*. *tabacum* Elongation Factor 1-alpha (*NtEF-1α*). Error bars indicate standard error. Colored asterisks indicate the statistical significance of the expression values relative to the wild-type controls with unpaired *t*-tests at *p* < 0.01.

Since nicotine formation is induced by JA treatment, we first examined the levels of *NtERF32*, *NtERF221* and *NtMYC2a* transcripts in various T2 overexpression lines with or without MeJA treatment by qRT-PCR. As shown in Figure 2, T2 lines overexpressing the *NtERF32*, *NtERF221*, and *NtMYC2a* genes under the control of 2X35S and GmUBI3 promoters showed only slight increases in transcript levels following MeJA treatment. This is not surprising since neither the 2X35S nor the GmUBI3 promoter are JA-responsive and there already existed high levels of transcript expression in the untreated plants. On the other hand, T2 transgenics expressing the three TF constructs under the control of the JA-responsive 4XGAG promoter were significantly (between 5- to 10-fold) induced by MeJA depending on the TF being expressed and the T2 line examined. It should be noted that the expression level of the three TFs was already significantly elevated relative to wild-type K326 plants in all the T2 transformants, regardless of the promoter used to drive expression, with the two constitutive promoters (2X35S and GmUBI3) being much higher than the K326 basal level, and higher than the expression driven by the 4XGAG promoter. This is likely due to the fact that the 4XGAG is barely activated in the absence of JA.

**Figure 3:**
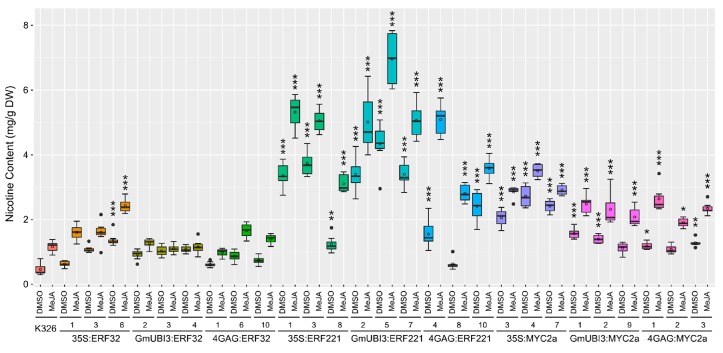
Quantification of nicotine in wild-type and transgenic tobacco by gas chromatography-mass spectrometry (GC-MS). Treatment with either 0.1% DMSO (control) or 100 µM MeJA was applied to 5-week-old wild-type or transgenic plantlets for 48 h. The leaf tissue was collected for alkaloid extraction and GC-MS was performed to quantify nicotine content. For each treatment, six to eight individuals were tested independently for each sample group. The distribution of nicotine level in each group is displayed with a boxplot. The upper and lower error bars represent the maximum and minimum values, solid dots represent outliers and the solid bar inside the box represents the median. Means are indicated with circles. Statistical analysis was performed with one-way analysis of variance (ANOVA) followed by Tukey’s honest significant difference (HSD) test for multiple pairwise comparisons. * indicates the level of significance based on the adjusted *p*-value between the wild-type and each transgenic group under the same treatment: *** *p* < 0.001, ** *p* < 0.01, * *p* < 0.05.

Our initial assessment of nicotine levels in the T1 generation of transgenics indicated that they had elevated nicotine contents relative to wild-type. To determine if creating homozygous T2 transgenic lines stabilized this phenotype and to more accurately quantify nicotine concentrations, GC-MS analysis was carried out on total alkaloids extracted from the leaf tissue of wild-type control plants and transgenic plants with or without MeJA treatment (Figure 3). The results are also summarized in Supplementary Table S1 as mean with standard deviation. Among different transgenic lines, T2 plants overexpressing *NtERF221* had on average the highest levels of nicotine concentration in their leaves both with and without phytohormonal treatment. In particular, the GmUBI3:ERF221 line #5 had almost 10-fold higher nicotine concentration (4.34 ± 0.66 mg/g) compared to the wild-type K326 (0.46 ± 0.17 mg/g) without MeJA elicitation, and about 6 times higher nicotine concentration (6.96 ± 0.95 mg/g over 1.16 ± 0.18 mg/g) when treated with MeJA. Approximately 2- to 5-fold increase or 1.5- to 2.5-fold increase of nicotine accumulation was observed in the transgenic lines overexpressing *NtMYC2a* compared to the basal level of nicotine in the wild-type with or without MeJA treatment (Figure 3 and Supplementary Table S1). The 35S:MYC2a lines had a little higher nicotine concentration than the GmUBI3:MYC2a and 4GAG: MYC2a lines did, yet it is not as high as that in most of the *NtERF221* overexpression lines. Compared to the JA-inducible 4XGAG promoter, both constitutive promoters gave higher nicotine production on average after MeJA treatment (Figure 3 and Supplementary Table S1). Intriguingly, nicotine concentration also showed a clear JA-inducible pattern in *NtERF221* and *NtMYC2a* constitutive overexpression lines, suggesting there may be other JA-mediated machinery or components for nicotine production that are independent of NtERF221. Unexpectedly, the overexpression of *NtERF32* exhibited the least effect on increasing nicotine concentration in transgenic lines (Figure 3 and Supplementary Table S1), even though TLC analysis showed significant elevation of nicotine accumulation in these lines (Figure 1b). Only one line of the 35:ERF32 transformants (line #6) showed the nicotine level about 2 times higher than the wild-type (Figure 3 and Supplementary Table S1).

### 3.2. Effects of Transcription Factors (TF) Overexpression on the Expression of Genes Involved in Nicotine Biosynthesis and Transport

**Figure 4:**
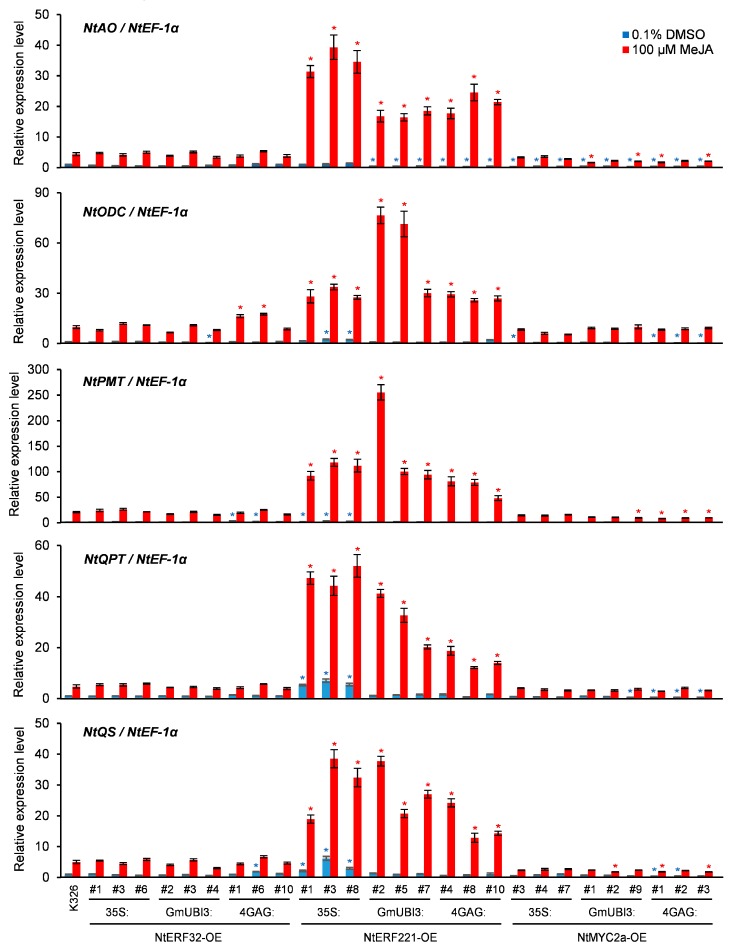
Expression levels of the structural genes that were upregulated by NtERF221 in wild-type and transgenic tobacco. Two-week-old wild-type or the transgenic tobacco seedlings overexpressing *NtERF32*, *NtERF221*, or *NtMCY2a* transcripts were treated with 0.1% DMSO (control) or 100 µM MeJA for 8 h before being collected for total RNA extraction. Transcript levels of *NtAO*, *NtODC*, *NtPMT*, *NtQPT*, and *NtQS* were measured by qRT-PCR, respectively. Expression level was represented as mean of relative expression values from three biological replicates (*n* = 3) normalized to *NtEF-1α*. Error bars indicate standard error. Colored asterisks indicate the statistical significance of the expression values relative to the wild-type controls with unpaired *t*-tests at *p* < 0.01.

To better understand the dynamics of TF overexpression and nicotine formation, we examined the expression of genes involved in nicotine biosynthesis and transport in the transgenic lines overexpressing *NtERF32*, *NtERF221*, and *NtMYC2a*. Transcript levels were measured in both wild-type and T2 transgenic seedlings with or without MeJA treatment. As shown in Figure 4, levels of *NtAO*, *NtODC*, *NtPMT*, *NtQPT*, and *NtQS* were slightly elevated in the *NtERF221* overexpressing lines compared to wild-type K326, as well as in some of the *NtERF32* and *NtMYC2a* overexpressing lines without MeJA treatment. However, following MeJA treatment, the *NtERF221* overexpressing lines had dramatically higher levels of induced transcripts for all five of these genes, while the *NtERF32* and *NtMYC2a* overexpressing lines had levels of induced expression similar to that of the wild-type (Figure 4). For example, the JA-induced transcript levels of *NtAO* in *NtERF221* overexpressing lines were 4 to 9 times higher than that in the wild-type. More than 10-fold increase in JA-induced expression of *NtPMT* and *NtQPT* in the *NtERF221* overexpressing lines than the wild type was observed (Figure 4).

In contrast, changes in the transcript level of several other nicotine biosynthetic genes (*NtA622*, *NtADC*, *NtBBL*, and *NtMPO*) or the *NtMATE* transporter gene were relatively small when compared to the wild-type with any of the transgenic tobacco overexpressing *NtERF32*, *NtERF221*, or *NtMYC2a* (Supplementary Figure S3). Nevertheless, all ten “nicotine biosynthetic genes” showed a clear JA-inducible expression pattern to different extents. These data indicated that NtERF221 is a very effective positive regulator for JA-mediated transactivation of several nicotine biosynthesis genes and its transactivation role may require other JA-inducible TFs or trans-acting factors. In addition, *NtA622*, *NtADC*, *NtBBL*, *NtMPO,* and *NtMATE*, whose expression are also JA-responsive, may not be under the transcriptional control of NtERF221 but rather a related NtERF family member.

### 3.3. Effects of the Overexpression of Biosynthetic Enzymes on Nicotine Accumulation

Previously, overexpression of *PMT* in *N*. *sylvestris* was reported to result in a significant increase in leaf nicotine level [67]. Similarly, silencing a tobacco-specific endogenous miRNA, nta-miRX27, which targets *QPT2*, led to an increase in the expression level of *QPT2* in the transgenic tobacco and an increase in nicotine biosynthesis [68]. Therefore, in addition to TFs we also examined the effect of the overexpression of several “nicotine genes” (i.e., *NtPMT1a*, *NtQPT2*, and *NtA622*) on nicotine accumulation in transgenic tobacco K326. To our surprise, we found that while significant increases in transcript levels were observed in the transgenic lines overexpressing *NtPMT1a*, *NtQPT2*, or *NtA622* (Supplementary Figures S4 and S5), we did not detect significant increases in nicotine content in any of the transgenic lines.

A possible reason for this result is that overexpression of a single enzyme in the nicotine biosynthesis pathway (Supplementary Figure S1) is likely not effective in uniformly increasing one or more rate-limiting substrates or precursors (e.g., putrescine, nicotinic acid mononucleotide, etc.) in the pathway required for enhancing downstream steps of nicotine biosynthesis, and thus, these biosynthetic intermediates remain rate limiting.

## 4. Discussion

We examined the potential of specifically targeting overexpression of TFs as a means of regulating nicotine biosynthesis in commercial tobacco. Previous studies have shown that genes within two genomic loci, *NIC1* and *NIC2* (originally named A and B), positively regulate nicotine formation [6,69,70,71,72] and that the *NIC2* locus was comprised of at least seven Group IX ERF family members [32]. Of these, *NtERF221* (originally designated *ORC1*) increased nicotine accumulation when overexpressed in tobacco [36] but there was no statistical difference from wild-type plants under limited analysis. Transient expression assay also identified the Group IX family members JAP1/NtERF10 and ORC1/NtERF221 as positive regulators of alkaloid production [30,31]. In the present study, we provide unequivocal evidence showing that T2 non-segregating transgenic plantlets overexpressing *NtERF221* effectively elevated nicotine accumulation in tobacco K326, even without MeJA elicitation.

NtERF221 and NtERF189 are closely related NIC2-locus ERFs within the Group IX NtERFs [35]. The role of NtERF189 in controlling stress responses and alkaloid production in cultured tobacco roots or cells is well documented, with the latter involving increased levels of nicotine biosynthetic enzyme gene expression via direct binding to the promoters of these genes [16,32,34,51]. Several GCC-box-like sequences were identified to be the binding sites for NtERF189 in the promoters of *NtPMT*, *NtQPT*, *NtODC,* and *NtMATE* genes [16,34]. In our transgenic *NtERF221* overexpressing lines, JA treatment greatly induced *NtAO*, *NtODC*, *NtPMT*, *NtQPT*, and *NtQS* transcript accumulation but not that of *NtA622* or *NtMATE* compared to wild-type (Figure 4 and Supplementary Figure S3), suggesting that NtERF221 and NtERF189 have highly similar yet distinct transcriptional activation profiles among the structural genes of nicotine biosynthesis. In addition, the JA-dependent pattern of the upregulation of the five nicotine biosynthesis genes in *NtERF221* transgenic lines suggested that the successful transactivation function of NtERF221 may still require other JA-responsive co-factors (e.g., members of the bHLH/MYC family). Nonetheless, the results from this work suggest that NtERF221 is a very effective TF target for enhancing nicotine biosynthesis in tobacco plants.

Transgenic tobacco lines overexpressing *JAP1*/*NtERF10* showed no significant increase in nicotine content [36] even though microarray-based analysis indicated that *JAP1*/*NtERF10* was among the top five most highly expressed genes following MeJA treatment of tobacco BY-2 cells [35]. In our study, we also observed that *NtERF10* overexpressing K326 tobacco exhibited little change in leaf nicotine concentration when compared to the wild-type control leaves (Supplementary Figure S6), although the transcript levels of *NtERF10* were much higher in the transgenic lines than the wild-type (Supplementary Figure S7). Why NtERF10 has little contribution to the regulation of nicotine biosynthesis in transgenic tobacco despite being able to bind JA-inducible promoters including those of the nicotine biosynthetic enzyme genes [37] is unclear but it suggests that other factors may be involved as well. Nevertheless, the substantial JA-responsive expression of *NtERF10* still makes it a very interesting topic as to which JA-dependent metabolic pathway(s) NtERF10 participates in.

Another two Group IX *ERF* genes, *NtERF32* and *NtERF121*, were also found to be rapidly induced by MeJA treatment, and overexpression of *NtERF32* greatly enhanced the transcript level of *NtPMT*, while RNAi suppression of *NtERF32* significantly reduced levels of total pyridine alkaloids, including nicotine in BY-2 cells [35]. In addition, *NtERF32* and *NtERF121*, previous called *ERF2* and *ERF5* respectively, have also been reported to be involved in wounding or pathogen-elicited defense responses [73,74,75]. Despite data suggesting these two NtERFs function as positive regulators of nicotine biosynthesis, the *NtERF121* overexpressing tobacco lines we generated in this work did not exhibit any appreciable increase in leaf nicotine accumulation (Supplementary Figure S6), although the transcript level of *NtERF121* also showed 30- to more than 200-fold increase in the transgenic lines compared to the wild-type (Supplementary Figure S7). The overexpression of *NtERF32* in this study did give increased nicotine level in some transgenic lines but its efficacy seemed limited in the transgenic tobacco K326 (Figure 3). In contrast to the previous result in tobacco BY-2 cells where overexpression or RNAi of *NtERF32* strongly up- or down-regulated the expression level genes involved in nicotine biosynthesis including *NtPMT* [35], no obvious upregulation of this kind could be observed in the transgenic tobacco overexpressing *NtERF32* (Figure 4 and Supplementary Figure S3). These results suggested that the NtERF32 may have diverse functions in other defensive responses while still partially participating in JA-induced nicotine or pyridine alkaloid biosynthesis.

Tobacco uses a canonical JA signal perception mechanism, which leads to the degradation of JAZ repressors to activate MYC transcription factors for the transactivation of the structural genes involved in nicotine biosynthesis, as first discovered in Arabidopsis [39,40,41,76,77] (Supplementary Figure S2). In *N*. *benthamiana*, two homologs of MYC2, NbbHLH1 and NbbHLH2, positively regulate the expression of *NbPMT* through binding to the G-box elements in the promoter region [33]. Suppression of the expression of *NbbHLH1* or *NbbHLH2* by RNAi substantially affected JA-induced expression of nicotine biosynthesis genes and the leaf nicotine accumulation in the transgenic tobacco plants, whereas only in *NbbHLH2* overexpression tobacco lines did the leaf nicotine content show a distinguishable increase [33]. Later, more G-box elements were identified from *NtPMT*, *NtQPT*, *NtA622,* and *NtMATE* as binding sites for NtMYC2 in tobacco [46,51]. Consistent with the finding in *N*. *benthamiana*, RNAi-mediated knock-down of *NtMYC2* genes in both transgenic tobacco hairy roots or BY-2 cells resulted in remarkable decrease of the expression levels of the structural genes such as *NtPMT*, *NtQPT,* and *NtA622*, as well as the level of nicotine production; whereas little or no increase in the expression levels of the structural genes could be found in *NtMYC2* overexpressing cell lines [46,51]. Despite the transactivation potential of NtMYC2 on a suite of nicotine biosynthesis genes, no conclusion could be made on whether the enhanced *NtMYC2* expression would lead to increased nicotine production in tobacco plants. Therefore, transgenic tobacco overexpressing *NtMYC2a* were generated to answer this question. On average, *NtMYC2a* transgenic lines showed a 2-fold increase in leaf nicotine content compared to wild-type leaves, but the effect of *NtMYC2a* overexpression on nicotine levels never reached that observed in the *NtERF221* overexpression lines (Figure 3 and Supplementary Table S1). Not surprisingly, transcript levels of the nicotine biosynthesis genes in these lines were not observed to be higher than control levels before or after MeJA treatment (Figure 4 and Supplementary Figure S3).

It is now well documented that JA treatment leads to the SCF^COI1^-dependent degradation of JAZ repressors at the 26S proteasome and release of NtMYC2 and related NtMYC TFs to bring about the activation of JA-responsive nicotine biosynthetic genes. Consistent with previous studies by Zhang et al. [46], overexpression of *NtMYC2* had little or no effect on the transactivation of nicotine structural genes in the presence or absence of exogenously added JA (Figure 4 and Supplementary Figure S3), underscoring the fact that for maximum transactivation, both AP2/ERF family and bHLH/MYC family TFs need to work synergistically to bring about JA-induced nicotine biosynthesis in tobacco [21,36,51]. More interestingly, a recent study in tomato discovered that overexpression of *MYC2* actually weakened JA responses in transgenic plants through a small group of JA-inducible MYC2-TARGETED bHLH (MTB) proteins. These MTBs act as transcriptional repressors of JA signaling by interference with the formation of the MYC2–MED25 interaction and competition with MYC2 for its target gene promoters [50]. Therefore, transgenic tobacco overexpressing *NtMYC2a* likely triggered increased levels of these negative feedback regulators simultaneously, which led to a less than desired level of nicotine production in these transgenics, since the MTB-like proteins are present in a wide range of plant species, including tobacco [50].

In conclusion, we have systematically examined the effects of manipulating levels of key TFs regulating or key enzymes involved in nicotine biosynthesis on the production and accumulation of this alkaloid in commercial tobacco. Our studies show that the targeted manipulation of NtERF221, a key transcription factor in the regulation of nicotine biosynthetic gene expression, is an effective strategy for the elevation of nicotine levels in the leaves of commercial tobacco for later use in preparation of extracts and cured leaf materials for use in reduced risk tobacco products and therapeutics. This work also provides additional details as to the consequences of TF manipulation on the overall mechanism of JA-mediated nicotine biosynthesis.

## Supplementary Materials

The following are available online at https://www.mdpi.com/2073-4425/10/11/930/s1, Table S1: Overview of nicotine content in wild-type and transgenic tobacco leaves, Figure S1: Biosynthetic pathways for nicotine and related pyridine alkaloids in tobacco (adapted from Dewey and Xie [4]), Figure S2: Model of JA-mediated transcriptional cascade for the activation of nicotine biosynthetic genes, Figure S3: Transcript levels of the structural genes involved in nicotine biosynthesis in wild-type and transgenic tobacco, Figure S4; TLC assay of the nicotine accumulation in wild-type, *NtPMT1a*, *NtQPT2* and *NtA622* transgenic tobacco, Figure S5: qRT-PCR analysis of the transcript levels of *NtPMT1a*, *NtQPT2*, and *NtA622* in wild-type and transgenic tobacco, Figure S6: TLC assay of the nicotine accumulation in wild-type, *NtERF10* and *NtERF121* transgenic tobacco, Figure S7: qRT-PCR verification of the transcript levels of *NtERF10* and *NtERF121* in wild-type and transgenic tobacco.

## References

[1] Kessler A., Baldwin I.T. (2002). Plant responses to insect herbivory: The emerging molecular analysis. Annu. Rev. Plant Biol..

[2] Kessler A., Baldwin I.T. (2004). Herbivore-induced plant vaccination. Part I. The orchestration of plant defenses in nature and their fitness consequences in the wild tobacco *Nicotiana attenuata*. Plant J..

[3] Steppuhn A., Gase K., Krock B., Halitschke R., Baldwin I.T. (2004). Nicotine’s defensive function in nature. PLoS Biol..

[4] Dewey R.E., Xie J. (2013). Molecular genetics of alkaloid biosynthesis in *Nicotiana tabacum*. Phytochemistry.

[5] Saitoh F., Noma M., Kawashima N. (1985). The Alkaloid Contents of 60 *Nicotiana* Species. Phytochemistry.

[6] Saunders J.W., Bush L.P. (1979). Nicotine Biosynthetic Enzyme Activities in *Nicotiana tabacum* L. Genotypes with Different Alkaloid Levels. Plant Physiol..

[7] Baldwin I.T. (1989). Mechanism of damage-induced alkaloid production in wild tobacco. J. Chem. Ecol..

[8] Baldwin I.T. (1998). Jasmonate-induced responses are costly but benefit plants under attack in native populations. Proc. Natl. Acad. Sci. USA.

[9] Baldwin I.T., Schmelz E.A., Ohnmeiss T.E. (1994). Wound-induced changes in root and shoot jasmonic acid pools correlate with induced nicotine synthesis in *Nicotiana sylvestris* spegazzini and comes. J. Chem. Ecol..

[10] Baldwin I.T. (1996). Methyl jasmonate-induced nicotine production in *Nicotiana attenuata*: Inducing defenses in the field without wounding. Entomol. Exp. Appl..

[11] Morita M., Shitan N., Sawada K., Van Montagu M.C.E., Inze D., Rischer H., Goossens A., Oksman-Caldentey K.M., Moriyama Y., Yazaki K. (2009). Vacuolar transport of nicotine is mediated by a multidrug and toxic compound extrusion (MATE) transporter in *Nicotiana tabacum*. Proc. Natl. Acad. Sci. USA.

[12] Shoji T., Inai K., Yazaki Y., Sato Y., Takase H., Shitan N., Yazaki K., Goto Y., Toyooka K., Matsuoka K. (2009). Multidrug and Toxic Compound Extrusion-Type Transporters Implicated in Vacuolar Sequestration of Nicotine in Tobacco Roots. Plant Physiol..

[13] Shitan N., Minami S., Morita M., Hayashida M., Ito S., Takanashi K., Omote H., Moriyama Y., Sugiyama A., Goossens A. (2014). Involvement of the Leaf-Specific Multidrug and Toxic Compound Extrusion (MATE) Transporter Nt-JAT2 in Vacuolar Sequestration of Nicotine in *Nicotiana tabacum*. PLoS ONE.

[14] Bush L., Hempfling P.W., Burton H., John W., Gorrod P.J. (1999). Chapter 2—Biosynthesis of nicotine and related compounds. Analytical Determination of Nicotine and Related Compounds and their Metabolites.

[15] Ziegler J., Facchini P.J. (2008). Alkaloid Biosynthesis: Metabolism and Trafficking. Annu. Rev. Plant Biol..

[16] Shoji T., Hashimoto T. (2011). Recruitment of a duplicated primary metabolism gene into the nicotine biosynthesis regulon in tobacco. Plant J..

[17] Hashimoto T., Yamada Y. (1994). Alkaloid Biogenesis: Molecular Aspects. Annu. Rev. Plant Biol..

[18] Imanishi S., Hashizume K., Nakakita M., Kojima H., Matsubayashi Y., Hashimoto T., Sakagami Y., Yamada Y., Nakamura K. (1998). Differential induction by methyl jasmonate of genes encoding ornithine decarboxylase and other enzymes involved in nicotine biosynthesis in tobacco cell cultures. Plant Mol. Biol..

[19] Riechers D.E., Timko M.P. (1999). Structure and expression of the gene family encoding putrescine N-methyltransferase in *Nicotiana tabacum*: New clues to the evolutionary origin of cultivated tobacco. Plant Mol. Biol..

[20] Bortolotti C., Cordeiro A., Alcazar R., Borrell A., Culianez-Macia F.A., Tiburcio A.F., Altabella T. (2004). Localization of arginine decarboxylase in tobacco plants. Physiol. Plant..

[21] Xu B.F., Timko M.P. (2004). Methyl jasmonate induced expression of the tobacco putrescine N-methyltransferase genes requires both G-box and GCC-motif elements. Plant Mol. Biol..

[22] Heim W.G., Sykes K.A., Hildreth S.B., Sun J., Lu R.H., Jelesko J.G. (2007). Cloning and characterization of a *Nicotiana tabacum* methylputrescine oxidase transcript. Phytochemistry.

[23] Katoh A., Shoji T., Hashimoto T. (2007). Molecular Cloning of N-methylputrescine Oxidase from Tobacco. Plant Cell Physiol..

[24] Sinclair S.J., Murphy K.J., Birch C.D., Hamill J.D. (2000). Molecular characterization of quinolinate phosphoribosyltransferase (QPRtase) in *Nicotiana*. Plant Mol. Biol..

[25] Katoh A., Uenohara K., Akita M., Hashimoto T. (2006). Early Steps in the Biosynthesis of NAD in Arabidopsis Start with Aspartate and Occur in the Plastid. Plant Physiol..

[26] Ryan S.M., Cane K.A., DeBoer K.D., Sinclair S.J., Brimblecombe R., Hamill J.D. (2012). Structure and expression of the quinolinate phosphoribosyltransferase (QPT) gene family in *Nicotiana*. Plant Sci..

[27] DeBoer K.D., Lye J.C., Aitken C.D., Su A.K.K., Hamill J.D. (2009). The A622 gene in *Nicotiana glauca* (tree tobacco): Evidence for a functional role in pyridine alkaloid synthesis. Plant Mol. Biol..

[28] Kajikawa M., Hirai N., Hashimoto T. (2009). A PIP-family protein is required for biosynthesis of tobacco alkaloids. Plant Mol. Biol..

[29] Kajikawa M., Shoji T., Kato A., Hashimoto T. (2011). Vacuole-Localized Berberine Bridge Enzyme-Like Proteins Are Required for a Late Step of Nicotine Biosynthesis in Tobacco. Plant Physiol..

[30] De Sutter V., Vanderhaeghen R., Tilleman S., Lammertyn F., Vanhoutte I., Karimi M., Inze D., Goossens A., Hilson P. (2005). Exploration of jasmonate signalling via automated and standardized transient expression assays in tobacco cells. Plant J..

[31] Rushton P.J., Bokowiec M.T., Han S.C., Zhang H.B., Brannock J.F., Chen X.F., Laudeman T.W., Timko M.P. (2008). Tobacco transcription factors: Novel insights into transcriptional regulation in the Solanaceae. Plant Physiol..

[32] Shoji T., Kajikawa M., Hashimoto T. (2010). Clustered transcription factor genes regulate nicotine biosynthesis in tobacco. Plant Cell.

[33] Todd A.T., Liu E., Polvi S.L., Pammett R.T., Page J.E. (2010). A functional genomics screen identifies diverse transcription factors that regulate alkaloid biosynthesis in *Nicotiana benthamiana*. Plant J..

[34] Shoji T., Hashimoto T. (2012). DNA-binding and transcriptional activation properties of tobacco NIC2-locus ERF189 and related transcription factors. Plant Biotechnol..

[35] Sears M.T., Zhang H.B., Rushton P.J., Wu M., Han S.C., Spano A.J., Timko M.P. (2014). NtERF32: A non-NIC2 locus AP2/ERF transcription factor required in jasmonate-inducible nicotine biosynthesis in tobacco. Plant Mol. Biol..

[36] DeBoer K.D., Tilleman S., Pauwels L., Vanden Bossche R., De Sutter V., Vanderhaeghen R., Hilson P., Hamill J.D., Goossens A. (2011). APETALA2/ETHYLENE RESPONSE FACTOR and basic helix-loop-helix tobacco transcription factors cooperatively mediate jasmonate-elicited nicotine biosynthesis. Plant J..

[37] Shoji T., Mishima M., Hashimoto T. (2013). Divergent DNA-Binding Specificities of a Group of ETHYLENE RESPONSE FACTOR Transcription Factors Involved in Plant Defense. Plant Physiol..

[38] Sheard L.B., Tan X., Mao H.B., Withers J., Ben-Nissan G., Hinds T.R., Kobayashi Y., Hsu F.F., Sharon M., Browse J. (2010). Jasmonate perception by inositol phosphate-potentiated COI1-JAZ co-receptor. Nature.

[39] Zhang F., Yao J., Ke J.Y., Zhang L., Lam V.Q., Xin X.F., Zhou X.E., Chen J., Brunzelle J., Griffin P.R. (2015). Structural basis of JAZ repression of MYC transcription factors in jasmonate signalling. Nature.

[40] Chini A., Fonseca S., Fernandez G., Adie B., Chico J.M., Lorenzo O., Garcia-Casado G., Lopez-Vidriero I., Lozano F.M., Ponce M.R. (2007). The JAZ family of repressors is the missing link in jasmonate signalling. Nature.

[41] Thines B., Katsir L., Melotto M., Niu Y., Mandaokar A., Liu G.H., Nomura K., He S.Y., Howe G.A., Browse J. (2007). JAZ repressor proteins are targets of the SCFCO11 complex during jasmonate signalling. Nature.

[42] Browse J. (2009). Jasmonate Passes Muster: A Receptor and Targets for the Defense Hormone. Annu. Rev. Plant Biol..

[43] Pauwels L., Barbero G.F., Geerinck J., Tilleman S., Grunewald W., Perez A.C., Chico J.M., Bossche R.V., Sewell J., Gil E. (2010). NINJA connects the co-repressor TOPLESS to jasmonate signalling. Nature.

[44] Chen R., Jiang H., Li L., Zhai Q., Qi L., Zhou W., Liu X., Li H., Zheng W., Sun J. (2012). The Arabidopsis mediator subunit MED25 differentially regulates jasmonate and abscisic acid signaling through interacting with the MYC2 and ABI5 transcription factors. Plant Cell.

[45] An C., Li L., Zhai Q., You Y., Deng L., Wu F., Chen R., Jiang H., Wang H., Chen Q. (2017). Mediator subunit MED25 links the jasmonate receptor to transcriptionally active chromatin. Proc. Natl. Acad. Sci. USA.

[46] Zhang H.-B., Bokowiec M.T., Rushton P.J., Han S.-C., Timko M.P. (2012). Tobacco Transcription Factors NtMYC2a and NtMYC2b Form Nuclear Complexes with the NtJAZ1 Repressor and Regulate Multiple Jasmonate-Inducible Steps in Nicotine Biosynthesis. Mol. Plant.

[47] Yang Y.P., Guo J., Yan P.C., Li Y.S., Liu K., Gao P., Zhao H.P., Chen Y.B., Wang Y.D., Timko M.P. (2015). Transcriptome Profiling Identified Multiple Jasmonate ZIM-Domain Proteins Involved in the Regulation of Alkaloid Biosynthesis in Tobacco BY-2 Cells. Plant Mol. Biol. Report..

[48] Chadick J.Z., Asturias F.J. (2005). Structure of eukaryotic Mediator complexes. Trends Biochem. Sci..

[49] Malik S., Roeder R.G. (2005). Dynamic regulation of pol II transcription by the mammalian Mediator complex. Trends Biochem. Sci..

[50] Liu Y., Du M., Deng L., Shen J., Fang M., Chen Q., Lu Y., Wang Q., Li C., Zhai Q. (2019). MYC2 Regulates the Termination of Jasmonate Signaling via an Autoregulatory Negative Feedback Loop. Plant Cell.

[51] Shoji T., Hashimoto T. (2011). Tobacco MYC2 Regulates Jasmonate-Inducible Nicotine Biosynthesis Genes Directly and By Way of the NIC2-Locus ERF Genes. Plant Cell Physiol..

[52] Shoji T., Hashimoto T. (2015). Stress-induced expression of NICOTINE2-locus genes and their homologs encoding Ethylene Response Factor transcription factors in tobacco. Phytochemistry.

[53] Voelckel C., Krugel T., Gase K., Heidrich N., van Dam N.M., Winz R., Baldwin I.T. (2001). Anti-sense expression of putrescine N-methyltransferase confirms defensive role of nicotine in *Nicotiana sylvestris* against Manduca sexta. Chemoecology.

[54] Chintapakorn Y., Hamill J.D. (2003). Antisense-mediated down-regulation of putrescine N-methyltransferase activity in transgenic *Nicotiana tabacum* L. can lead to elevated levels of anatabine at the expense of nicotine. Plant Mol. Biol..

[55] Wang P., Zeng J., Liang Z.F., Miao Z.Q., Sun X.F., Tang K.X. (2009). Silencing of PMT expression caused a surge of anatabine accumulation in tobacco. Mol. Biol. Rep..

[56] DeBoer K.D., Dalton H.L., Edward F.J., Hamill J.D. (2011). RNAi-mediated down-regulation of ornithine decarboxylase (ODC) leads to reduced nicotine and increased anatabine levels in transgenic *Nicotiana tabacum* L. Phytochemistry.

[57] Dalton H.L., Blomstedt C.K., Neale A.D., Gleadow R., DeBoer K.D., Hamill J.D. (2016). Effects of down-regulating ornithine decarboxylase upon putrescine-associated metabolism and growth in *Nicotiana tabacum* L. J. Exp. Bot..

[58] Farsalinos K.E., Polosa R. (2014). Safety evaluation and risk assessment of electronic cigarettes as tobacco cigarette substitutes: A systematic review. Ther. Adv. Drug Saf..

[59] Glasser A.M., Collins L., Pearson J.L., Abudayyeh H., Niaura R.S., Abrams D.B., Villanti A.C. (2017). Overview of Electronic Nicotine Delivery Systems: A Systematic Review. Am. J. Prev. Med..

[60] Quik M., Perez X.A., Bordia T. (2012). Nicotine as a potential neuroprotective agent for Parkinson’s disease. Mov. Disord..

[61] Quik M., Boyd J.T., Bordia T., Perez X. (2019). Potential Therapeutic Application for Nicotinic Receptor Drugs in Movement Disorders. Nicotine Tob. Res..

[62] Murashige T., Skoog F. (1962). A Revised Medium for Rapid Growth and Bio Assays with Tobacco Tissue Cultures. Physiol. Plant..

[63] Horsch R.B., Fry J.E., Hoffmann N.L., Eichholtz D., Rogers S.G., Fraley R.T. (1985). A Simple and General-Method for Transferring Genes into Plants. Science.

[64] Schmidt G.W., Delaney S.K. (2010). Stable internal reference genes for normalization of real-time RT-PCR in tobacco (*Nicotiana tabacum*) during development and abiotic stress. Mol. Genet. Genom..

[65] Goossens A., Hakkinen S.T., Laakso I., Seppanen-Laakso T., Biondi S., De Sutter V., Lammertyn F., Nuutila A.M., Soderlund H., Zabeau M. (2003). A functional genomics approach toward the understanding of secondary metabolism in plant cells. Proc. Natl. Acad. Sci. USA.

[66] Hernandez-Garcia C.M., Bouchard R.A., Rushton P.J., Jones M.L., Chen X., Timko M.P., Finer J.J. (2010). High level transgenic expression of soybean (Glycine max) GmERF and Gmubi gene promoters isolated by a novel promoter analysis pipeline. BMC Plant Biol..

[67] Sato F., Hashimoto T., Hachiya A., Tamura K., Choi K.B., Morishige T., Fujimoto H., Yamada Y. (2001). Metabolic engineering of plant alkaloid biosynthesis. Proc. Natl. Acad. Sci. USA.

[68] Li F.F., Wang W.D., Zhao N., Xiao B.G., Cao P.J., Wu X.F., Ye C.Y., Shen E.H., Qiu J., Zhu Q.H. (2015). Regulation of Nicotine Biosynthesis by an Endogenous Target Mimicry of MicroRNA in Tobacco. Plant Physiol..

[69] Legg P.D., Collins G.B. (1971). Inheritance of Per Cent Total Alkaloids in *Nicotiana tabacum* L. 2. Genetic Effects of 2 Loci in Burley 21 X La Burley 21 Populations. Can. J. Genet. Cytol..

[70] Hibi N., Higashiguchi S., Hashimoto T., Yamada Y. (1994). Gene Expression in Tobacco Low-Nicotine Mutants. Plant Cell.

[71] Cane K.A., Mayer M., Lidgett A.J., Michael A.J., Hamill J.D. (2005). Molecular analysis of alkaloid metabolism in AABB v. aabb genotype *Nicotiana tabacum* in response to wounding of aerial tissues and methyl jasmonate treatment of cultured roots. Funct. Plant Biol..

[72] Kidd S.K., Melillo A.A., Lu R.H., Reed D.G., Kuno N., Uchida K., Furuya M., Jelesko J.G. (2006). The A and B Loci in Tobacco Regulate a Network of Stress Response Genes, Few of which are Associated with Nicotine Biosynthesis. Plant Mol. Biol..

[73] Ohme-Takagi M., Shinshi H. (1995). Ethylene-Inducible DNA Binding Proteins That Interact with an Ethylene-Responsive Element. Plant Cell.

[74] Fischer U., Dröge-Laser W. (2004). Overexpression of NtERF5, a New Member of the Tobacco Ethylene Response Transcription Factor Family Enhances Resistance to Tobacco mosaic virus. Mol. Plant-Microbe Interact..

[75] Nakano T., Nishiuchi T., Suzuki K., Fujimura T., Shinshi H. (2006). Studies on Transcriptional Regulation of Endogenous Genes by ERF2 Transcription Factor in Tobacco Cells. Plant Cell Physiol..

[76] Katsir L., Chung H.S., Koo A.J., Howe G.A. (2008). Jasmonate signaling: A conserved mechanism of hormone sensing. Curr. Opin. Plant Biol..

[77] De Geyter N., Gholami A., Goormachtig S., Goossens A. (2012). Transcriptional machineries in jasmonate-elicited plant secondary metabolism. Trends Plant Sci..

