# Supplementary material for: Increased Leaf Nicotine Content by Targeting Transcription Factor Gene Expression in Commercial Flue-Cured Tobacco (Nicotiana tabacum L.)
Source: Genes (Basel). 2019 Nov 14;10(11):930. doi: 10.3390/genes10110930 (PMC6896058; doi:10.3390/genes10110930)
Supplement: Supplementary file 1 [file genes-10-00930-s001.zip › Supplementary Files/Table S1.docx]

Table S1. Overview of nicotine content in wild-type and transgenic tobacco leaves.

| **Sample Name** | **Nicotine (mg/g DW)*** | |
| --- | --- | --- |
|  | **DMSO** | **MeJA** |
| WT | 0.46 ± 0.17 | 1.16 ± 0.18 |
| 35S:ERF32-1 | 0.63 ± 0.09 | 1.61 ± 0.23 |
| 35S:ERF32-3 | 1.09 ± 0.12 | 1.61 ± 0.35 |
| 35S:ERF32-6 | 1.38 ± 0.21 | 2.43 ± 0.26 |
| GmUBI3:ERF32-2 | 0.92 ± 1.15 | 1.26 ± 0.16 |
| GmUBI3:ERF32-3 | 1.03 ± 0.17 | 1.10 ± 0.14 |
| GmUBI3:ERF32-4 | 1.08 ± 0.10 | 1.15 ± 0.21 |
| 4GAG:ERF32-1 | 0.61 ± 0.08 | 0.98 ± 0.12 |
| 4GAG:ERF32-6 | 0.87 ± 0.15 | 1.66 ± 0.23 |
| 4GAG:ERF32-10 | 0.74 ± 0.12 | 1.40 ± 0.15 |
| 35S:ERF221-1 | 3.35 ± 0.40 | 5.32 ± 0.53 |
| 35S:ERF221-3 | 3.74 ± 0.38 | 5.05 ± 0.37 |
| 35S:ERF221-8 | 1.24 ± 0.25 | 3.11 ± 0.27 |
| GmUBI3:ERF221-2 | 3.40 ± 0.53 | 5.01 ± 0.95 |
| GmUBI3:ERF221-5 | 4.34 ± 0.66 | 6.96 ± 0.95 |
| GmUBI3:ERF221-7 | 3.40 ± 0.36 | 5.06 ± 0.57 |
| 4GAG:ERF221-4 | 1.55 ± 0.42 | 5.09 ± 0.51 |
| 4GAG:ERF221-8 | 0.63 ± 0.19 | 2.81 ± 0.28 |
| 4GAG:ERF221-10 | 2.41 ± 0.45 | 3.58 ± 0.32 |
| 35S:MYC2a-3 | 2.06 ± 0.28 | 2.85 ± 0.19 |
| 35S:MYC2a-4 | 2.72 ± 0.34 | 3.50 ± 0.24 |
| 35S:MYC2a-7 | 2.42 ± 0.20 | 2.91 ± 0.17 |
| GmUBI3:MYC2a-1 | 1.57 ± 0.15 | 2.48 ± 0.32 |
| GmUBI3:MYC2a-2 | 1.40 ± 0.14 | 2.32 ± 0.53 |
| GmUBI3:MYC2a-9 | 1.12 ± 0.17 | 2.08 ± 0.31 |
| 4GAG:MYC2a-1 | 1.19 ± 0.11 | 2.64 ± 0.42 |
| 4GAG:MYC2a-2 | 1.08 ± 0.14 | 1.90 ± 0.14 |
| 4GAG:MYC2a-3 | 1.28 ± 0.13 | 2.35 ± 0.20 |

* Numbers are the mean ± standard deviation of the GC-MS measurements.

DW, dry weight.
