# Supplementary figures and images for: Increased Leaf Nicotine Content by Targeting Transcription Factor Gene Expression in Commercial Flue-Cured Tobacco (Nicotiana tabacum L.)
Source: Genes (Basel). 2019 Nov 14;10(11):930. doi: 10.3390/genes10110930 (PMC6896058; doi:10.3390/genes10110930)

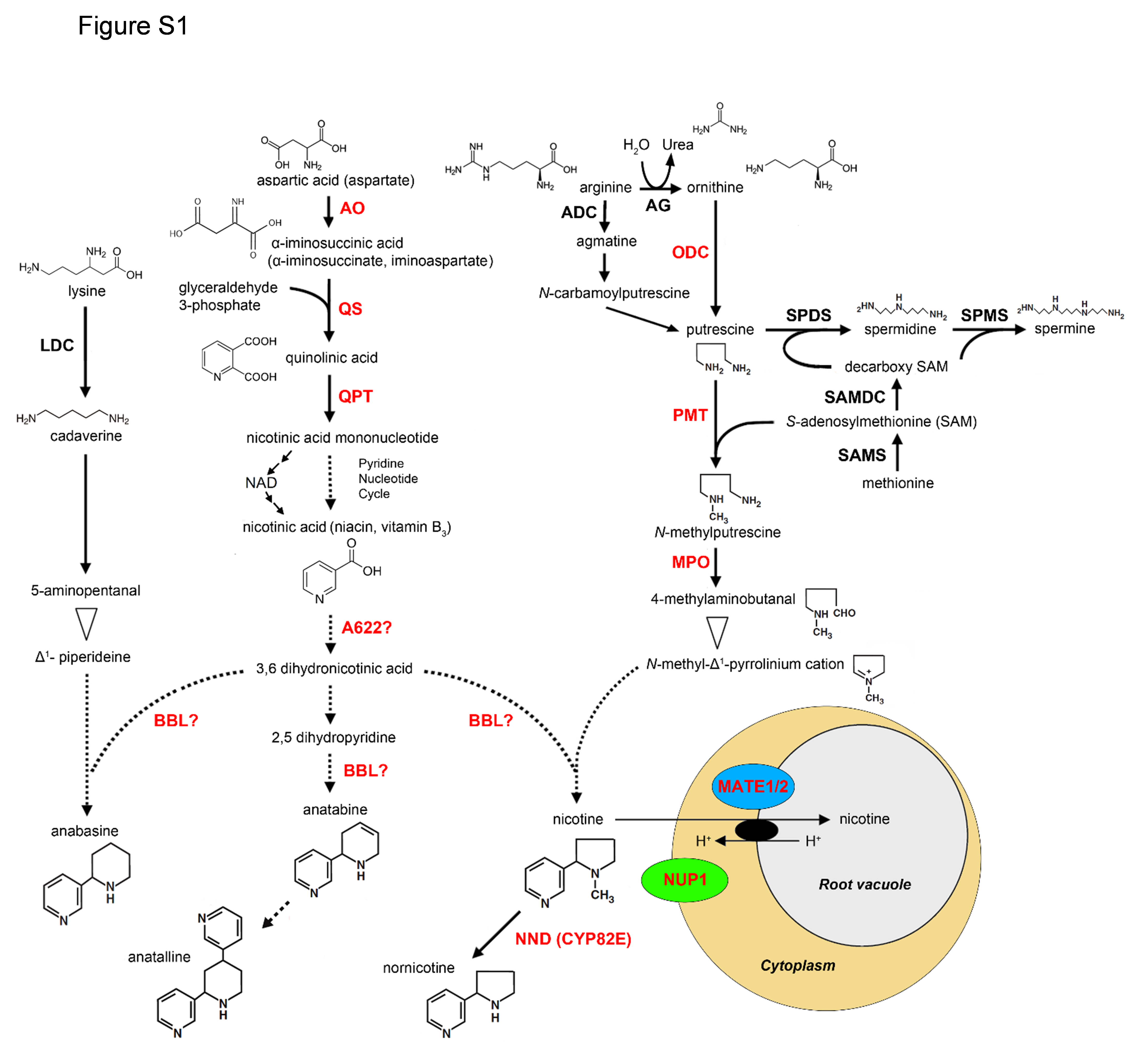

Supplement: Supplementary file 1 [file genes-10-00930-s001.zip › Supplementary Files/Figure S1.tif]

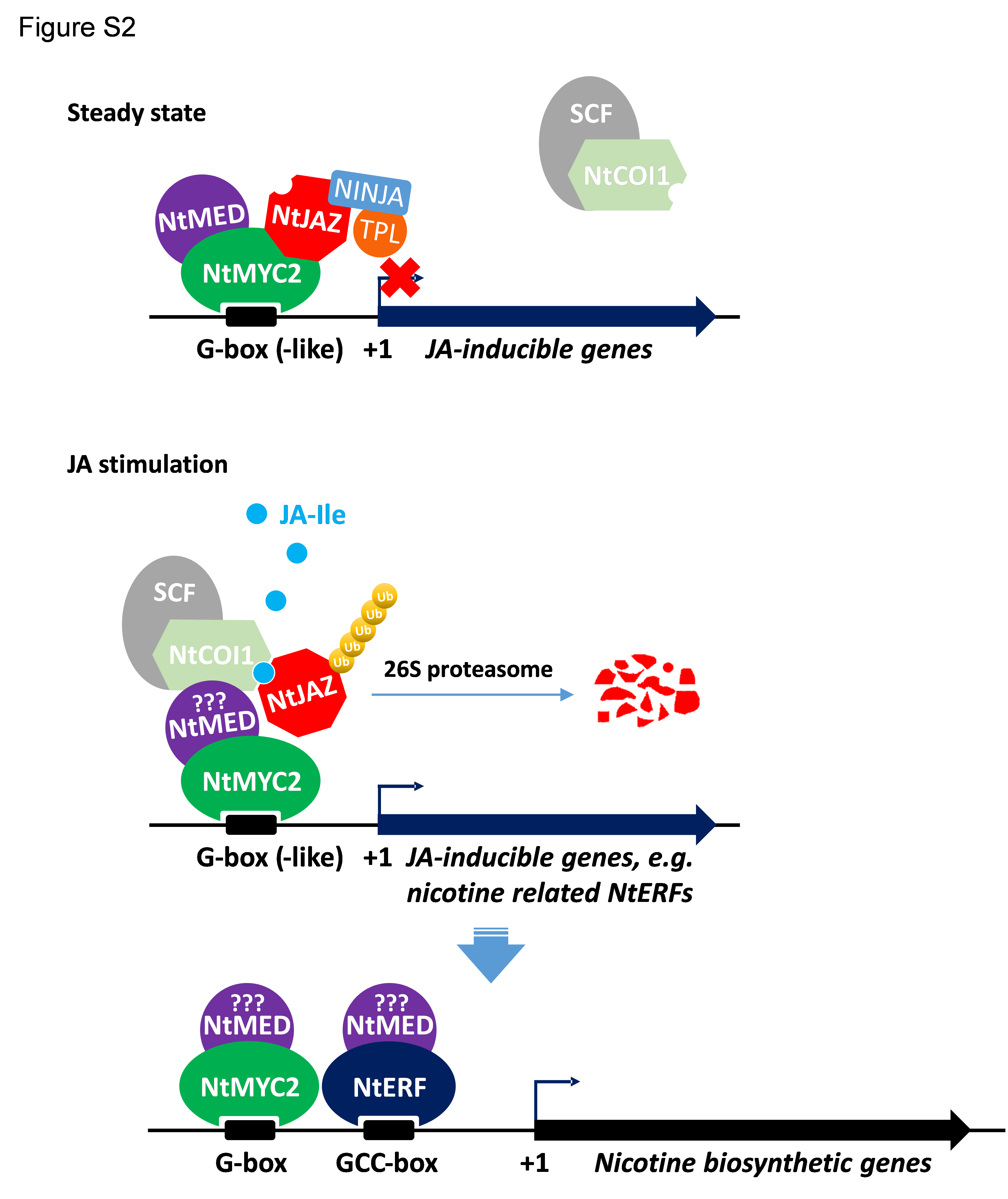

Supplement: Supplementary file 1 [file genes-10-00930-s001.zip › Supplementary Files/Figure S2.tif]

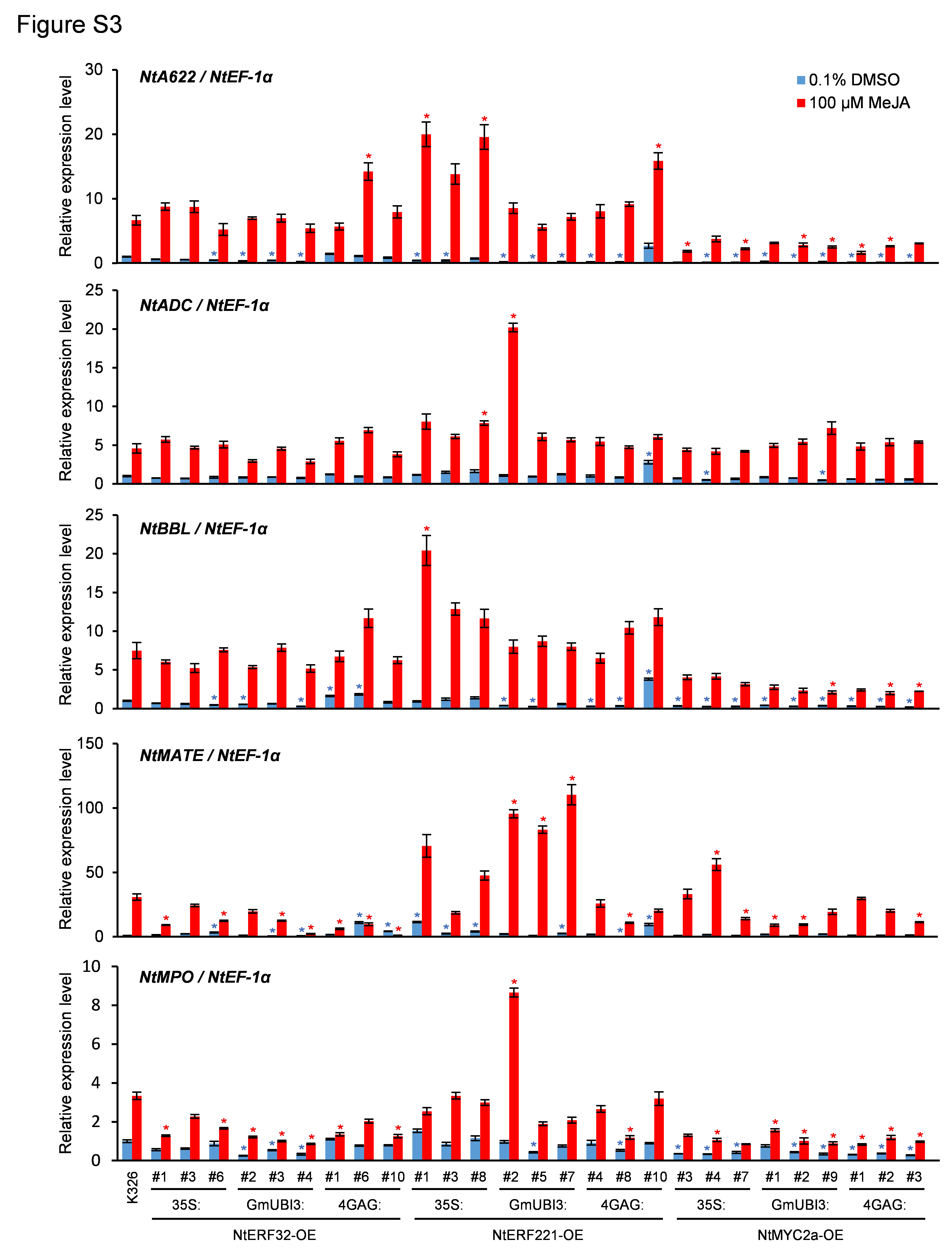

Supplement: Supplementary file 1 [file genes-10-00930-s001.zip › Supplementary Files/Figure S3.tif]

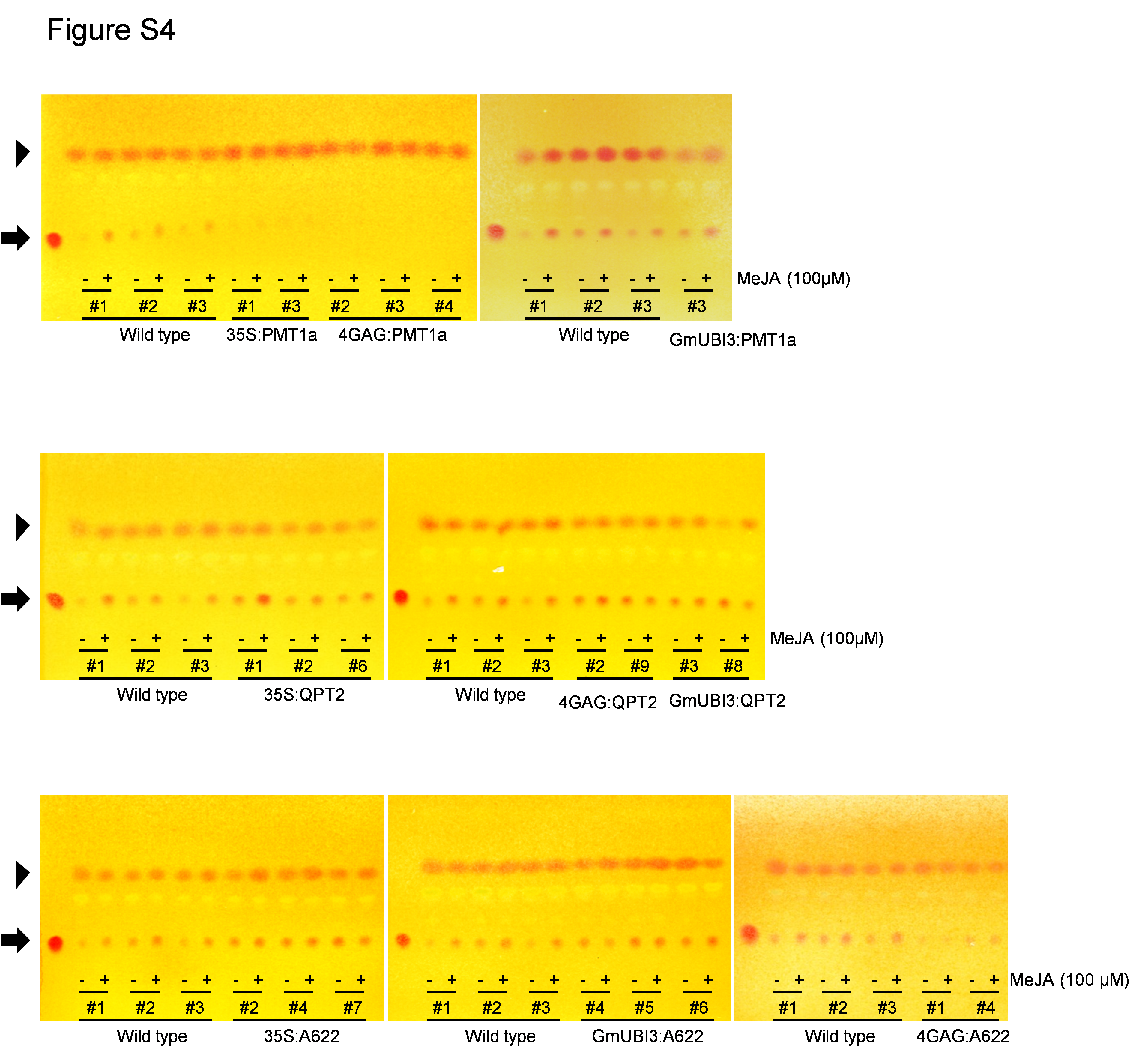

Supplement: Supplementary file 1 [file genes-10-00930-s001.zip › Supplementary Files/Figure S4.tif]

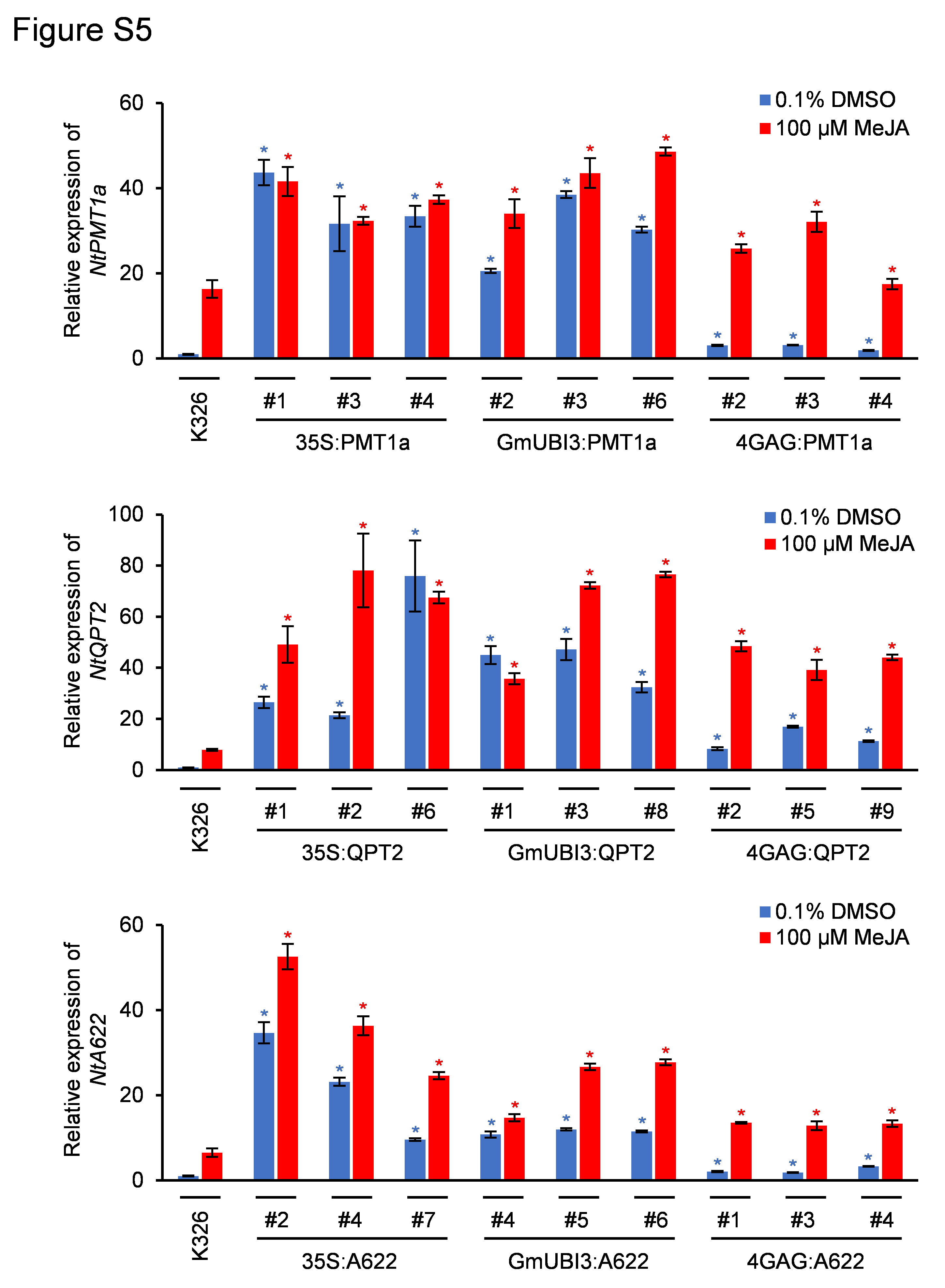

Supplement: Supplementary file 1 [file genes-10-00930-s001.zip › Supplementary Files/Figure S5.tif]

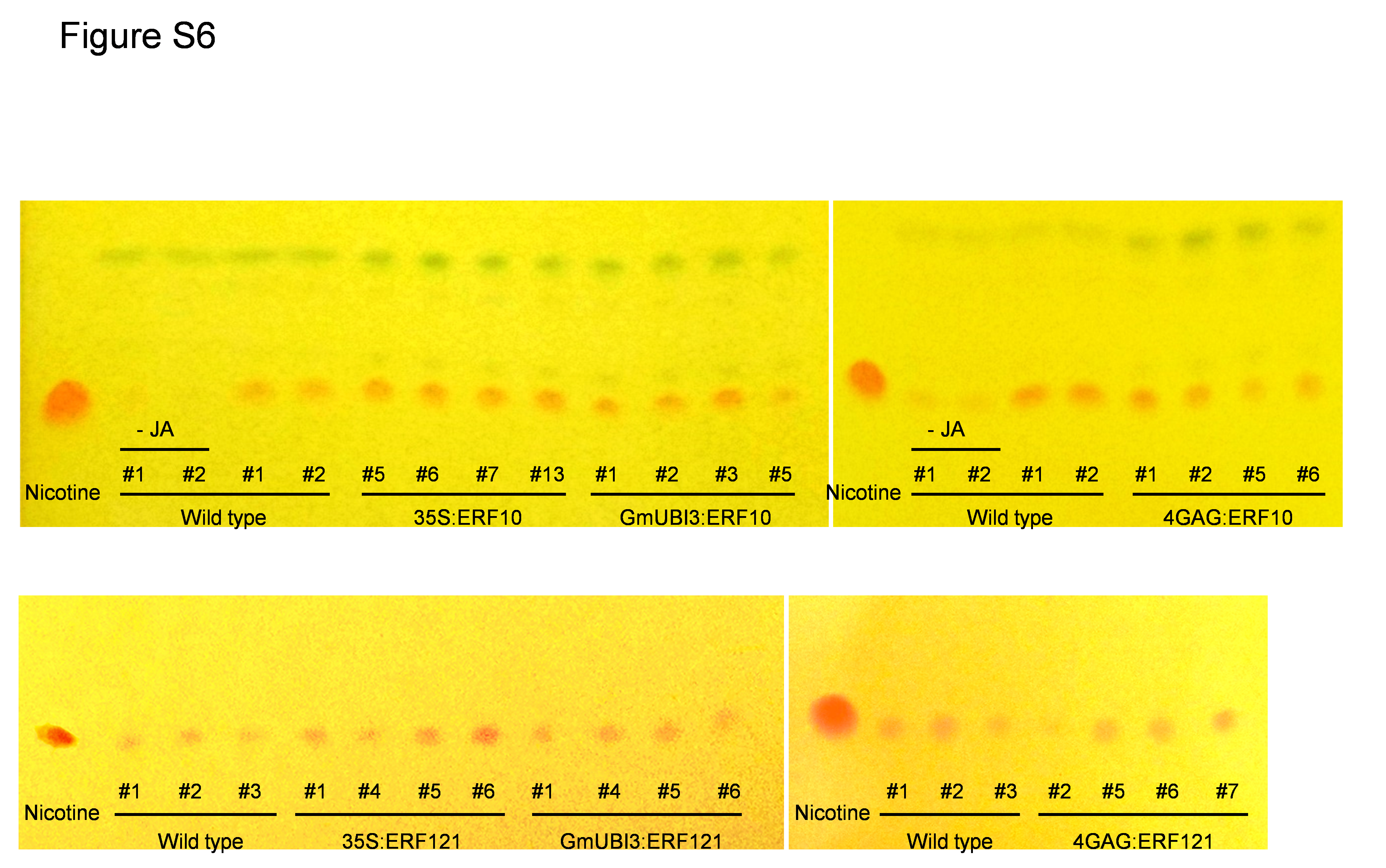

Supplement: Supplementary file 1 [file genes-10-00930-s001.zip › Supplementary Files/Figure S6.tif]

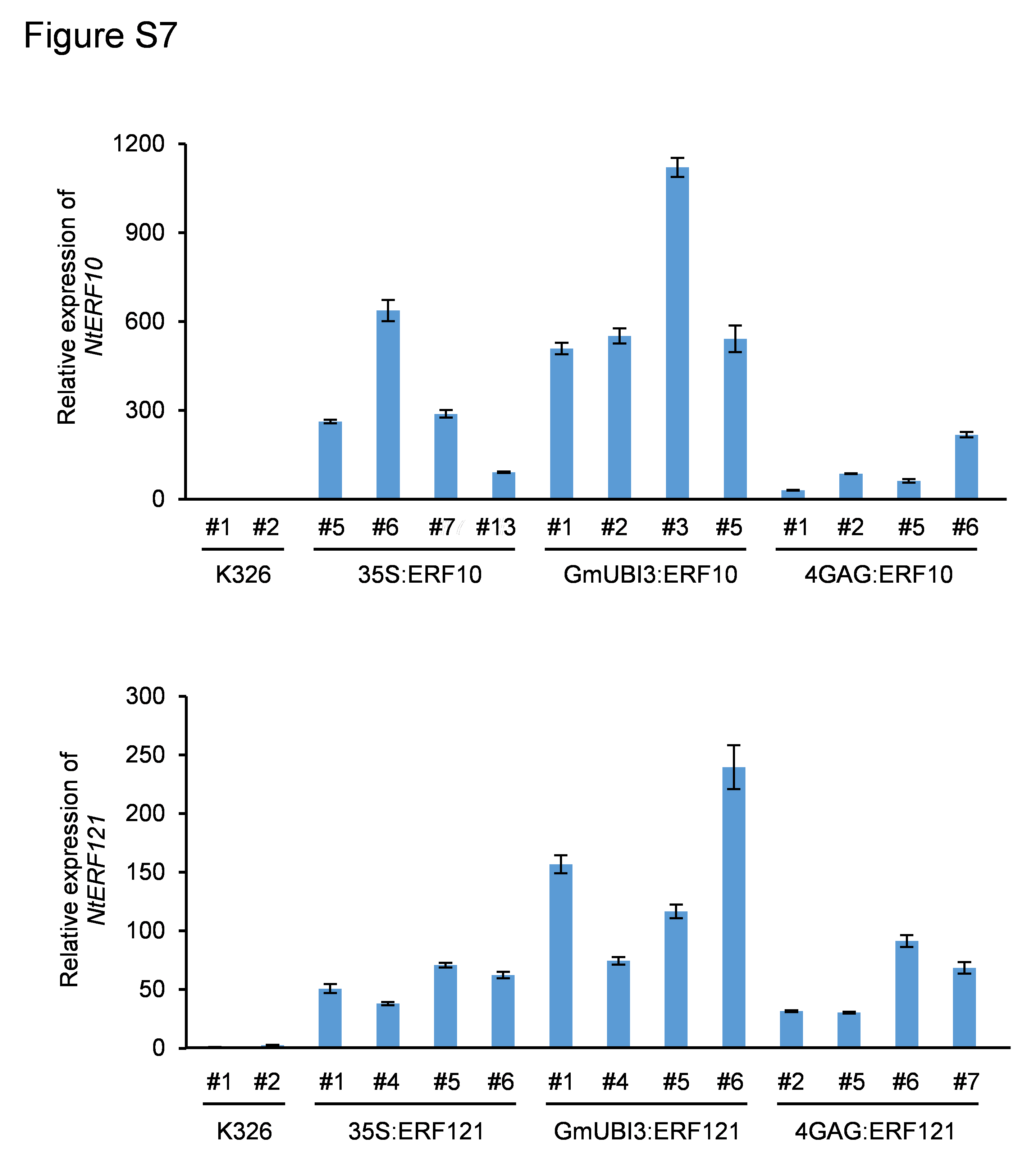

Supplement: Supplementary file 1 [file genes-10-00930-s001.zip › Supplementary Files/Figure S7.tif]
